# Cell-based therapy in thin endometrium and Asherman syndrome

**DOI:** 10.1186/s13287-021-02698-8

**Published:** 2022-01-28

**Authors:** Nastaran Gharibeh, Leili Aghebati-Maleki, Javad Madani, Ramin Pourakbari, Mehdi Yousefi, Javad Ahmadian Heris

**Affiliations:** 1 Student’s Research Committee, Tabriz University of Medical Sciences, Tabriz, Iran; 2 Stem Cell Research Center, Tabriz University of Medical Sciences, Tabriz, Iran; 3 Immunology Research Center, Tabriz University of Medical Sciences, Tabriz, Iran; 4 Department of Allergy and Clinical Immunology, Pediatric Hospital, Tabriz University of Medical Sciences, Tabriz, Iran; 5 Department of Immunology, School of Medicine, Tabriz University of Medical Sciences, Tabriz, Iran

**Keywords:** Cell therapy, Endometrium, Asherman syndrome

## Abstract

Numerous treatment strategies have so far been proposed for treating refractory thin endometrium either without or with the Asherman syndrome. Inconsistency in the improvement of endometrial thickness is a common limitation of such therapies including tamoxifen citrate as an ovulation induction agent, acupuncture, long-term pentoxifylline and tocopherol or tocopherol only, low-dose human chorionic gonadotropin during endometrial preparation, aspirin, luteal gonadotropin-releasing hormone agonist supplementation, and extended estrogen therapy. Recently, cell therapy has been proposed as an ideal alternative for endometrium regeneration, including the employment of stem cells, platelet-rich plasma, and growth factors as therapeutic agents. The mechanisms of action of cell therapy include the cytokine induction, growth factor production, natural killer cell activity reduction, Th17 and Th1 decrease, and Treg cell and Th2 increase. Since cell therapy is personalized, dynamic, interactive, and specific and could be an effective strategy. Despite its promising nature, further research is required for improving the procedure and the safety of this strategy. These methods and their results are discussed in this article.

## Introduction

The route through which thin endometrium reduces the chance of pregnancy is not well-known. However, two proposed hypotheses discussing the fact that the embryo could be close to the basal layer, which is rich in reactive oxygen species and thus detrimental to the implantation and development of the embryo [1]. One or two among three women who undergo in vitro fertilization and embryo transfer are affected by a thin endometrium (> 7 mm). One attributed cause could be the malfunctioning of endometrial stem/progenitor cells of the incompetent thin endometrium [2]. Satisfactory endometrium growth is an essential factor for successful implantation since low implantation rates have been reported to be associated with thin endometrium. Further, a thin endometrium is reported to be a defective cause of endometrial development. Endometrium recovery in thin endometrium patients is time-consuming although low-dose aspirin treatment or estrogen therapy has been assessed in this regard [3].

The development of the human endometrium is typically observed through transvaginal ultrasound in assisted reproductive technology cycles. A well-established marker for the receptivity of the uterus is the thickness of the endometrium [4]. The minimal cutoff endometrial thickness proposed for a successful embryo transfer is 7 mm, even though there exists no standardized value for diagnosing a thin endometrium [5]. In addition, values above 9 mm could predict greater implantation rates. In fact, persistent thin endometrium in the reproductive cycle could be related to lower rates of implantation as well as increased rates of miscarriages [6]. Consequently, many researchers have sought to identify therapeutic modalities capable of improving endometrial receptivity and growth in endometrium-thin women [7]. Although numerous approaches (i.e., granulocyte colony-stimulating factor, sildenafil, or low-dose aspirin) have been attempted clinically, their results are inconclusive [8]. On the other hand, an important outcome of endometrial trauma is the intrauterine adhesion (IUA), resulting in the partial or full obstruction of the cervical canal or uterine cavity. Asherman syndrome (AS) defines the IUA, which is characterized by infertility, recurrent abortion, hypomenorrhea, pelvic pain, or menopause [9]. Operation procedures could generally lead to IUA through inducing artificial traumas to the uterine cavity. For instance, hysteromyomectomy, cesarean section, and curettage could injure the endometrial basal layer. Moreover, infections such as tuberculosis can cause chronic endometrial inflammation, which is in fact an inducer of adhesion [9].

The incidence rate of the AS is approximately 1.5% [10], and the hysteroscopy adhesion lysis is regarded as the current prevalent treatment approach for this condition [11]. Nevertheless, susceptibility to anomalous placenta development and preterm delivery exists in patients following adhesion lysis due to impaired endometrial angiogenesis and metabolism [12]. It is essential to prevent adhesion in the uterine cavity post-invasive operations since two-thirds of AS-suffering women have experienced post-abortion/miscarriage curettage [13]. This could be performed through placing an intrauterine device [14]/Foley’s catheter balloon/hyaluronic acid in the cavity of the uterus or employing conjugated estrogen treatment for facilitating the recovery of the endometrium [15].

**Fig. 1:**
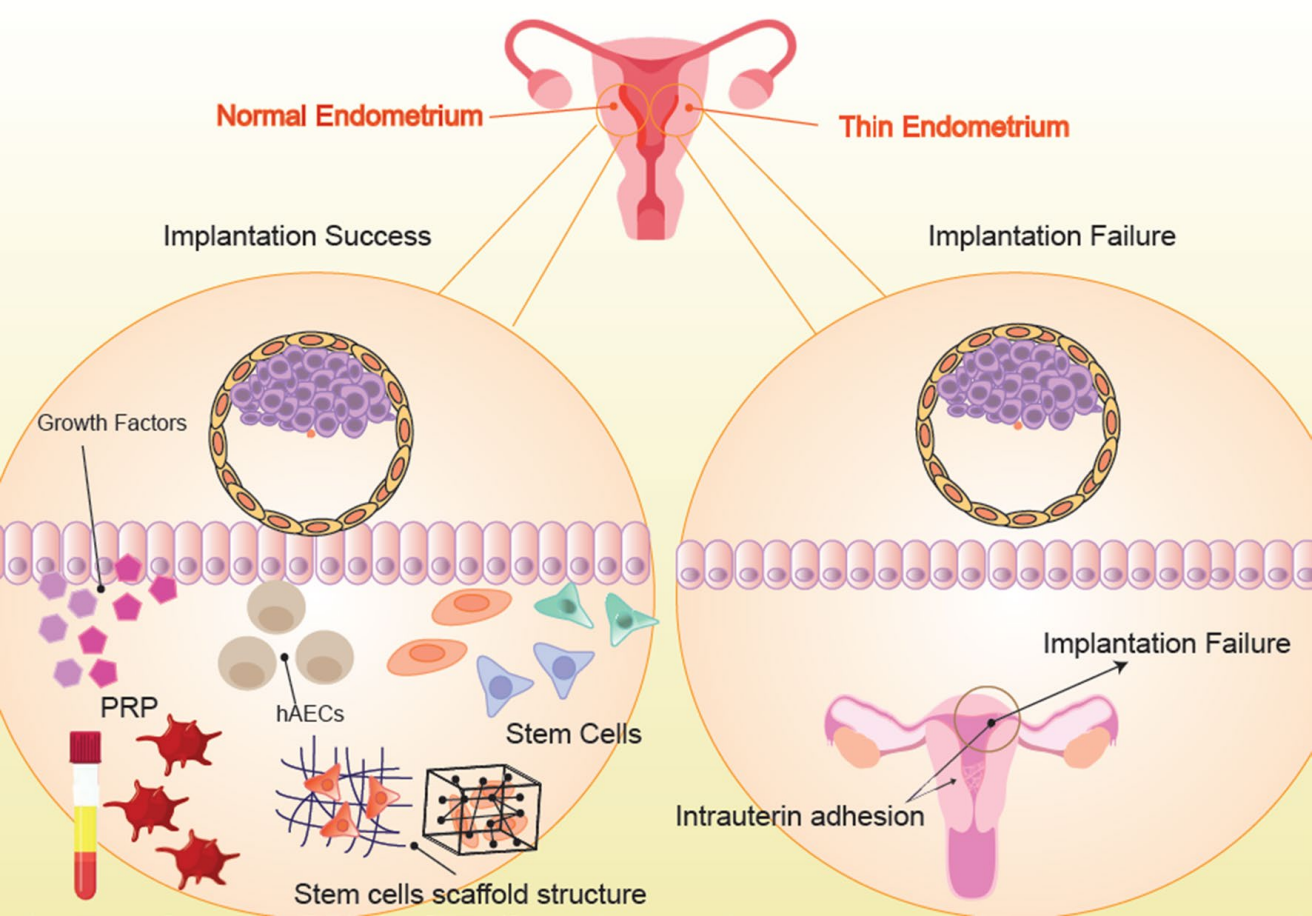
On the right side of the figure, a patient with a thin endometrium is illustrated who has had implantation failure. On the left side of the figure, different types of cell therapy, which lead to the thickness of the patient's endometrium and subsequently success in pregnancy, are shown

Cell therapy, as one of the most effective fields of translational medicine, is an interdisciplinary field encompassing regenerative medicine, transplantation biology, biomaterials, molecular biology, tissue engineering, immunology, stem cell biology, and clinical research [14]. Cell-based therapy possesses the potential to turn into a novel therapeutic platform for treating a wide range of clinical disorders. Two major examples of cell-based therapeutics include bone marrow transplantation and blood transfusions [16]. Although recombinant genetic engineering has been effective in producing manifold therapeutics (e.g., human insulin and erythropoietin), such treatments are not capable of entirely correcting or reversing disease states [17]. This is because disease processes mostly involve changes in the multifaceted interactions of different cell components rather than a deficiency in a single protein. Under such circumstances, cell-based therapy might prove more effective through the provision of an individualized, interactive, and dynamic therapeutic approach responding to the pathophysiological condition of the patient [18]. The most recent approaches for the treatment of thin endometrium and the AS will be thoroughly reviewed in the following sections (Fig. 1).

## Platelet-rich plasma (PRP)

As an available option, autologous PRP has been prominent for its safety and has been employed since the 1970s. The safety of therapy through the infusion of PRP is attributed to the fact that it is prepared from autologous blood, which itself is obtained from a peripheral vein. More importantly, preparation procedures are painless for the patient, affordable, and convenient. Since PRP releases numerous chemokines, cytokines, and growth factors stored in the alpha granules of platelets, it is capable of improving tissue regeneration [19]. Processes affected by these molecules include angiogenesis, the remodeling of the extracellular matrix, as well as the differentiation, proliferation, and recruitment of stem cells [20]. Therefore, PRP is applicable in many regenerative medicine fields such as dermatology, ophthalmology, plastic surgery, and orthopedics for promoting tissue growth and repair [21–23].

However, immunogenic and transmission reactions could be prevented since PRP is derived from autologous blood. Moreover, PRP treatment is regarded as safe according to the results of the treatment of thousands of individuals with PRP following oral-maxillary surgery [24]. More precisely, infection and other adverse effects are reported to be rare in patients. Overall, there exist several factors that could potentially determine the success of this treatment, including the selection of patients and preparation of proper cell [25].

So far, there has been limited information with regard to the potential that the application of PRP might hold in the restoration of damaged endometrium [26]. However, it has been reported that treatment with PRP led to the enhanced thickness of the endometrium (ultrasonography-approved), elevated rates of pregnancy (clinically-approved), and raised live-birth rates [27]. Nonetheless, it is unclear whether such improvements were directly related to PRP treatment. Further, the inadequacy of the experimental evidence could be attributed to the lack of objective validity. In an in vivo study, treatment with autologous PRP resulted in enhanced endometrium regeneration in female rats, which was confirmed by real-time polymerase chain reaction assays related to the expression of endometrial factors [28]. In another study on murine AS models without or with the infusion of human PRP, Kim et al. assessed the capability for regenerating the endometrium and the mating outcomes. However, data could not distinctively imply that the donor’s hormonal profile could exert effects on the therapeutic potential of his/her PRP. PRPs from separate donors showed comparable results, emphasizing that the PRP is capable of restoring damaged endometrium. Importantly, the quantification of successful pregnancy and implantation outcomes were evaluated as well. The treatment group with PRP considerably enhanced the IS number and supported the capability of carrying the pregnancy to term, resulting in an 83.3% live-birth in PRP-treated AS mice while other AS mice failed the delivery [29].

In an experiment, Wang et al. [30] investigated 20 women suffering from thin endometrium and undergoing failed embryo implantation under conventional estrogen therapy, resulting in the cancellation of the cycle and low pregnancy. Following PRP infusion, the majority of patients showed effective pregnancy and expansion of endometrium. Ultrasonography was used to evaluate the thickness of the endometrium, followed by exploring the effects of PRP on biological functions. Accordingly, PRP was capable of promoting the migration and proliferation of endometrial mesenchymal stem cells (EnMSCs), subsequently differentiating into endometrial cells. Moreover, it could be stated that PRP stimulated adhesion to Matrigel and the spreading of cells. Additional examples of PRP treatment are provided in Table 1.

**Table 1:** All cell therapies performed to date on thin endometrium and Asherman syndrome

Type of cell therapy	Authors	Model	Result	References
*Platelet-rich plasma (PRP)*				
PRP	Kim et al.	Murine model of Asherman’s syndrome	Human PRP helps down-regulate the expression of fibrosis-related factors, restores uterine function of impaired uterine horns, and improves implantation outcomes following endometrial injury in mice, enabling full-term delivery and improving the rate of live-births	[29]
PRP + BMSCs	Zhou et al.	Injured Rat Uterus	PRP up-regulates IL-10 production	[31]
Activated PRP	Aghajanova et al.	In vitro	MMP1, MMP3, MMP7, and MMP26 were increased by aPRP	[32]
PRP	Marini et al.	In vitro	PRP treatment significantly down regulated the expression of pro-inflammatory genes	[33]
PRP	Zadehmodarres et al.	Thin endometrium patients	It seems that PRP was effective for endometrial growth in patient with thin endometrium	[26]
PRP	Chang et al.	women undergoing in vitro fertilization (IVF)	Platelet-rich plasma (PRP) was able to promote the endometrial growth and improve pregnancy outcome of patients with thin endometrium	[34]
PRP	Kim et al.	Thin endometrium patients	The use of autologous PRP improved the implantation, pregnancy, and live birth rates of the patients with refractory thin endometrium	[27]
*Growth factor*				
G-CSF	Gleicher	Thin endometrium patients	This cohort study is supportive of the effectiveness of G-CSF in expanding chronically unresponsive endometria	[35]
G-CSF	Check et al.	Thin endometrium patients	Improvement in the endometrial thickness in women with consistently thin endometria	[36]
G-CSF	Kunicki et al.	Thin endometrium in women undergoing in vitro fertilization	Infusion of G-CSF leads to the improvement of endometrium thickness after 72 h	[37]
G-CSF	Shah et al.	Thin endometrium in women undergoing in vitro fertilization	infusion of G-CSF to achieve significant increase in the endometrial thickness with higher successful pregnancy rate among infertile women under-going IVF-ET cycles with a history of a persistently thin endometrium	[38]
G-CSF	Xu et al.	Patients were diagnosed with thin endometrium	Significantly higher embryo implantation and clinical pregnancy rates were observed in the G-CSF group compared with the control group	[39]
G-CSF	Tehraninejad et al.	Thin endometrium patients	G-CSF may increase endometrial thickness in the small group of patients who had no choice except cycle cancellation or surrogacy	[40]
*Stem cells*				
MSCs	Kilic et al.	Rat	MSCs is added to estrogen, regeneration of endometrium is stimulated	[41]
BMDSCs	Feryal Alawadhi et al.	Mice	After BMDSC transplant, the rate of fertility improves in Asherman’s Syndrome mice, indicating a BMDSC functional role in uterine regeneration	[42]
Autologous SCs	Singh et al.	Human	Menstrual reconstruction in 5 out of 6 cases revealed the role of autologous stem cell transplantation in endometrial regeneration	[43]
BMSCs	Jing et al.	Rat	The results of this study using rat model showed that BMSCs can play a significant role in reconstruction of thin endometrium by locating in the endometrium, differentiating into numerous cells, and being immunomodulatory	[44]
eMSCs	Ulrich et al.	Human	eMSC provides an available alternative origin of MSC for use in cell-based therapies. It becomes evident that eMSC inhabits in the endometrium have ceased after a woman's fertile years	[45]
hUCMSCs	Tang et al.	Rat	This study has demonstrated that transplantation of hUCMSCs can efficiently reduce the fibrosis area of endometrium, also enhance glandular count and upgrade proliferation of endometrial cells in IUA rat	[46]
BMSCs	Wang et al.	Rat	BMSCs transplantation had an impressive effect on regenerating of the injured endometrium probably via promoting the expression of ER and PR in rat models	[47]
Autologous CD133 + BMDSCs	Santamaria et al.	Human	Increase in the congestion of mature vessel and the severity and period of menses in the first 3 months are the advantages of the CD133 + BMDSCs therapy. In the AS and EA, the thickness of Endometrium increased approximately from 4.3 mm to 6.7 mm	[48]
menSCs	Jichun Tan et al.	Human	The transplantation of Autologous menSCs considerably rise endometrial thickness (ET) for women with severe AS	[49]
hESP cells	Irene Cervelló et al.	Human	The mesenchymal origin of hESP confirmed by their ability to differentiate in vitro into osteocytes and adipocytes. Eventually, after transplanted under renal capsule of NOD-SCID mice they have displayed the potency to generate human endometrium	[50]
Autologous adipose derived stem cells (ADSCs)	Sudoma et al.	Human	ADSCs subendometrial introduction led to endometrial thickness increase, 13 pregnancies occurred and 9 healthy babies were born	[51]
uterus derived mesenchymal stem cells and their exosomes	Saribas et al.	Rat	It was shown that proliferation and vascularization increased and fibrosis decreased in uterus as a result of MSC and exosome treatments	[52]
Autologous bone marrow-derived stem cell	Singh et al.	Human	Intrauterine stem cell treatment is a promising novel approach for refractory cases of AS and EA	[53]
Autologous adipose derived stem cells (ADSCs)	Yotsumoto et al.	Mice	ADSCs may be a useful therapeutic strategy to improve fertility of women with thin endometrium	[54]
*Human amniotic epithelial cells (hAEC)*				
Human amniotic epithelial cells (hAEC)	Song et al.	Rat	This study revealed that hESCs along with collagen scaffolds could notably support function recovery and uterine repair in a rat model of intense uterine injury	[55]
Human amniotic epithelial cells (hAEC)	Ouyang et al.	Rat	These results indicate that hAECs transplantation promote endometrial regeneration and the restoration of fertility in rat model of IUA	[56]
Human amniotic mesenchymal stromal cell	Gan et al.	Rat	hAMSC transplantation promotes endometrial regeneration after injury in IUA rat models, possibly due to immunomodulatory properties	[57]
Human amniotic epithelial cells	Li et al.	Mice	hAECs have the potential to repair the uterus after injury, providing a new strategy for the prevention and treatment of Asherman syndrome	[58]
Human amniotic epithelial cells	Bai et al.	Rat	hAEC transplantation could inhibit the progression of fibrosis and promote proliferation and angiogenesis in IUA rat models	[59]
*Nanostructured scaffold*				
Collagen scaffold with collagen-binding human basic fibroblast growth factor	Conforti et al.	Rat	Transplantation of collagen scaffold with collagen-binding human basic fibroblast growth factor promote Regeneration of uterine horns	[60]
Collagen scaffold with umbilical cord MSCs	Xin et al.	Human	Transplantation of collagen scaffold with umbilical cord MSCs improves endometrial thickness	[61]
Collagen scaffold with BM-MNCs	Ballios et al.	Human	Transplantation of collagen scaffold with BM-MNCs promote functional endometrium reconstruction via downregulating ΔNp63 expression	[62]
Collagen scaffold with BM-MSCs	Dolmans et al.	Rat	Transplantation of collagen scaffold with BM-MSCs improve the level of bFGF, IGF-1, TGFβ1 and VEGF in blood vessels	[63]
Collagen scaffold with BM-MSCs	Eliopoulos et al.	Rat	Transplantation of collagen scaffold with BM-MSCs promote uterus regeneration	[64]

## Growth factors

As naturally occurring substances, growth factors (GFs) have the potential to stimulate cellular differentiation, wound healing, and cell proliferation [65] and are typically regarded as intercellular signaling molecules. For instance, hormones or cytokines are capable of binding to particular surface receptors of target cells, which frequently promote cellular maturation and differentiation differently. Epidermal growth factor (EGF) is capable of enhancing osteogenic differentiation, whereas vascular endothelial and fibroblast GFs are capable of stimulating angiogenesis (differentiation of blood vessels) [66]. As a hematopoietic growth factor, granulocyte-colony-stimulating factor (G-CSF) has been demonstrated to be effective in non-hematopoietic cells such as the endometrium [67]. After the successful treatment of four individuals, it was postulated that intrauterine G-CSF may play a direct role in the promotion of endometrial growth [68]. A study on thin-endometrium patients revealed that intrauterine G-CSF administered 6–12 h before human chorionic gonadotropin (HCG) trigger could significantly improve endometrial thickness, resulting in an overall pregnancy rate of 19.1% [35].

In this regard, Lucena et al. [69] successfully treated one patient while Check et al. failed to do so. Following treatment, the rate of cycle cancelation caused by thin endometrium was considerably lower (69.39% self-controlled group versus 48.75% control group vs. 17.5% treatment group, *p* < 0.05), with a trend towards greater pregnancy and implantation rates [70]. In another study by Kunicki et al. [37], intrauterine G-CSF (300 μg) was administered to patients with a 7 mm EMT 6–12 h before HCG trigger, leading to a considerable improvement in EMT after 72 h, with a pregnancy rate of 18.9%. In a recent study, Barad et al. administered intrauterine G-CSF to FET or IVF patients regardless of the thickness of the endometrium. Furthermore, in another study, a 300 μg/cm^3^ dose of G-CSF was given to the study group on the day of the HCG trigger, indicating no improvements in the thickness of the endometrium [71]. Similarly, intrauterine G-CSF was administered to thin lining-possessing patients (< 8 mm) who were resistant to treatment with vaginal sildenafil or estradiol. The treatment was also expanded to patients with repeated failure of implantation who had an EMT > 8 mm [38]. In a study, G-CSF (300 μg) was administered to the endometrial cavity of all 231 patients 10 days post-vaginal sildenafil and oral estradiol, causing a considerable improvement in EMT and a pregnancy rate of 38.07% [72]. Further examples of GF administration are provided in Table 1.

## Stem cells therapy

**Fig. 2:**
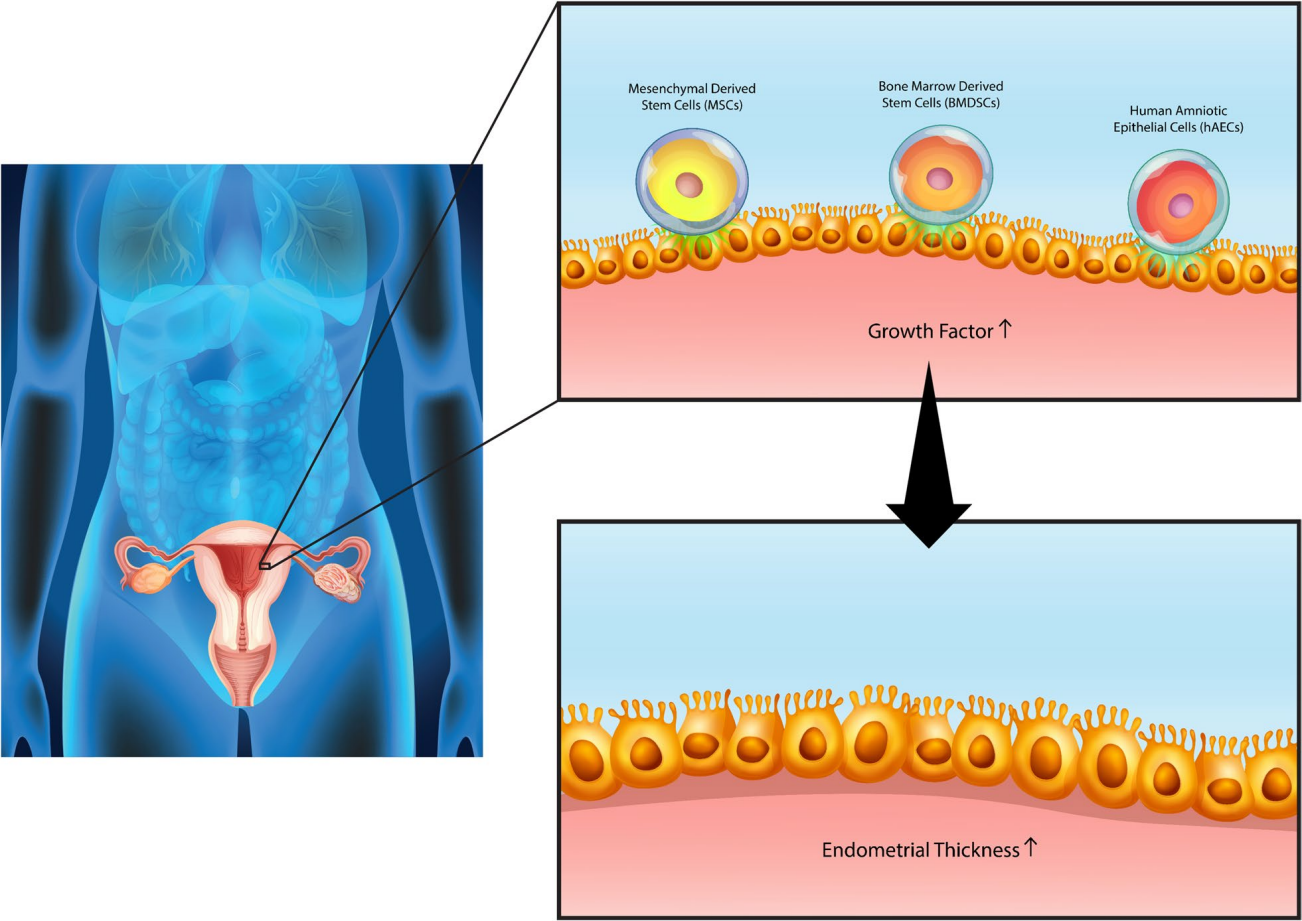
Schematic of thickening of the endometrium by stem cells with the mechanism of increase in growth factors in the target area

Stem cell therapy has been recognized as an efficient therapeutic approach among numerous types of cell therapy [73]. Stem cells are capable of dividing into pluripotent and multipotent stem cells, and several studies have enumerated both advantages and disadvantages associated with their utilization [74]. It has also been reported that it is feasible to employ stem cell therapy as a therapeutic approach for treating infertility-related diseases such as thin endometrium (Fig. 2). Numerous clinical, but challenging, trials have been performed in this regard. Overall, it seems likely that resolving issues associated with stem cell therapy could allow their application as an efficient therapeutic approach for patients suffering from infertility [75].

Similar to the majority of treatments, cell therapy could be accompanied by side effects, particularly in stem cell therapy [76]. For instance, prior to using stem cells, they should be grown for several months after they are harvested from embryos. Further, painful procedures are experienced when the aim is to obtain adult stem cells, especially from bone marrow. Moreover, stem cell treatments neither have been proven yet, nor encountered low rates of rejection [77]. Some examples of the application of stem cell therapy are summarized in Table 1.

### Mesenchymal derived stem cells (MSCs)

MSCs, a type of adult stem cells, could be harvested from various tissues including bone marrow, umbilical cord, menstrual blood, endometrial tissue, adipose tissue, and the like [78]. Given the capacity of self-renewal and differentiation potentials, the emerging research has regarded MSCs as attractive candidates for cell therapy in regenerative medicine [79]. Such reactivity could reflect the tissue of origin since dissimilar sensitivity to inductive bioactive molecules in culture is exhibited by MSCs isolated from different tissues. Well-known examples include adult marrow-derived MSCs, which are frequently employed as a standard type of MSCs. Conditions which induce marrow-derived MSCs differ from those of fat-derived MSCs, which could be attributed to the presence of different microenvironments in the tissue side of the vasculature within which the pericytes reside [80]. Tissue regeneration has experienced considerable progress since the application of MSCs [81]. Nonetheless, extremely functional in vitro assays have validated the encouraging regenerative potentials of MSCs. Protective effects exerted by MSCs following allogeneic transplantation have been reported in several injured models such as damaged, neural, myocardial, hepatic, cartilage, and bone tissues [82]. It is rather recognized that the therapeutic effects of MSCs are mainly due to their immunomodulatory function, which is performed along with anti-inflammatory effects through the regulation of lymphocytes of both adaptive and innate immune systems. Further, it has been established that MSCs are capable of regulating the immune responses in numerous diseases [83]. In fact, MSCs are capable of regulating the function and proliferation of T cells, balancing the activity of Th2 and Th1, up-regulating Tregs functions, suppressing the functions of B cells, inhibiting the function and proliferation of NK cells, and preventing the activation and maturation of dendritic cells [84]. Moreover, MSCs are capable of stimulating proliferation and the secretion of cytokines in innate lymphoid cells, which are a novel family of lymphocyte-like cells playing significant roles in the innate defenses against pathogens. The regulation of the immunomodulatory functions of MSCs is performed with regard to the inflammatory conditions of their microenvironment [85]. The intensity and type of the inflammatory stimuli that are presented on MSCs determine MSC plasticity in immunomodulation. As an example, MSCs are capable of suppressing the polarization of Th17 and Th1 and promoting the polarization of Th2 in graft-versus-host disease [86]. Moreover, MSCs are capable of inhibiting Th2-dominant allergy through the inhibition of the production of IL-13 and IL-4. MSCs contribute to the fibrotic process or immunosuppressive effects under chronic and acute inflammatory conditions, respectively. Therefore, MSCs could be regarded as a feasible and flexible strategy to treat several diseases, according to their immunomodulatory characteristics [87]. However, MSCs function deteriorates with age, which might be associated with the loss of tissue homeostasis, resulting in aging-related diseases and the malfunction of organs. Zhao et al. demonstrated that injecting bone marrow mesenchymal stem cells (BMSCs) into the uterine cavities of rats resulted in a thicker endometrium as well as the upregulation of vimentin and cytokeratin as marker proteins of endometrial cells. Resultantly, directly infusing BMSCs could protect the thin endometrium rats against cell damage, in addition to promoting endometrial cell regeneration. Post-BMSC transplantation, the expression of LIF and integrin αβ3 represented a considerable increase. It has been well established that LIF and integrin as the regulators of the endometrial function are the markers of the receptivity of the endometrium and possess significant parts in embryo implantation [88]. In another study [44], it was reported that the MSCs improved endometrium thickness probably via their migration and immunomodulatory properties. Table 1 provides additional examples of MSCs in the endometrium.

## MSC-derived extracellular vesicles (MSC-EVs)

On the other hand, recent studies have also focused on the investigation of exosomes secreted from mesenchymal stem cells. Exosomes are active paracrine components with a high potential for repairing damaged tissue. Exosomes include many paracrine factors responsible for regeneration and angiogenesis [52]. MSC-EVs play a critical role in treating reproductive diseases such as the AS. EVs are lipid bilayer complexes that function as mediators by transferring multiple molecules to recipient cells such as proteins, microRNAs, lipids, and cytokines. Although a consensus has been reached on the mechanisms underlying MSC-EVs, several theories have been proposed, including promoting angiogenesis, anti-fibrosis, immunomodulation, and anti-oxidative stress levels. Moreover, numerous questions need to be fully clarified before the application of MSC-EVs in the clinic, including standardized purification and identification methods, appropriate storage and transportation systems, determined cargo for large-scale generation, and safety issues. In addition, limited yield is one of the main problems restraining the wide-spreading application of MSC-EVs. Generally, MSC-EVs have exhibited their potentials in regenerative medicine not only for their propensity ability originated from the parent cells but also for the higher biology stability and lower immunogenicity as compared to MSCs [89].

### Bone marrow-derived stem cells (BMDSCs)

Being able to travel to distant organs, BMDSCs make a contribution to the regeneration and repair of tissues [90]. Since BMDSCs exist in both murine and human endometrium, it could be inferred that they have the potential for serving as a reparative cell source for the reproductive tract [91]. Regional signals of injury probably play a significant part in mobilizing BMDSCs to injured tissues. In fact, it is demonstrated that ischemia/reperfusion injury in the uterus enhances the migration and engraftment of BMDSCs in the endometrium [92].

Alawadhi et al. reported that by the third estrous cycle after BM transplantation, female mice were bred for three months. Nine out of ten mice were placed in the BM transplant group, whereas only three mice were conceived in the non-BM transplant group (Chi-square *p* = 0.0225). On the other hand, 10/10 mice were conceived in the control group, within which there was no uterine injury. The mean litter size in the BM transplant, the non-BM transplant, and control groups was 6.361.4, 5.364.0, and 7.062.0, respectively. The results indicated no significant differences between groups (*p* = 0.05) and in the time to conception between groups [42]. Further examples in this regard are presented in Table 1.

### Human amniotic epithelial cells (hAECs)

As the potential stem cell source, hAECs are isolated from the amniotic membrane, which is in contact with the amniotic fluid and is the closest layer to the fetus. The immunomodulatory effect of hAECs on adaptive and innate immune cells has been reported by many studies. In addition, they are capable of differentiating into numerous cells with mesoderm and ectoderm origin, including neural cells, pancreatic cells, hepatocytes, adipocytes, cardiomyocytes, and myocytes. Further, hAECs are extremely able to suppress the proliferation of B cells and inhibit the migration of neutrophils and macrophages [57]. Moreover, hAECs inhibit the activation of CD4+ T cells and decrease the proinflammatory cytokine production of CD4+ T cells. It has been reported that hAECs considerably enhance proliferative cell nuclear antigen (PCNA), which is responsible for accurate DNA duplication [93]. Comparing samples of the endometrium from reproductive-age women, Niklaus et al. revealed that PCNA was most abundant at the proliferative phase in both epithelial and stroma tissues. However, it reduced at the secretory phase expression in murine endometrium, suggesting the fact that hAECs may be capable of improving endometrial proliferation. Being mostly expressed throughout the proliferative phase and menstrual period, vascular endothelial growth factor (VEGF) is associated with maintaining and formulating microvessels, as well as reconstructing the endometrial tissue [94]. Chen et al. demonstrated that hysteroscopy adhesion lysis in combination with hormone replacement therapy in IUA patients significantly enhanced the expression of endometrial VEGF and MVD. Moreover, the ones with better curative effects did have greater expression of VEGF and denser microvessels as compared to patients whose responses to the treatment were poor [95]. Zhou et al. found that hAECs were capable of increasing the expression of VEGF in IUA models, demonstrating the angiogenesis potential of hAECs which may improve the recovery of endometrial injuries. Oestrogen receptor (ER), as a nuclear transcription factor, combined with estrogen is capable of promoting the proliferation and metabolism in endometrial cells [96]. In the repaired endometrium of allogeneic UCMSC-treated individuals, the expression of ER is considerably enhanced [97]. In line with this study, hAECs were reported to be able to increase the expression of ER in damaged murine endometrium. Overall, ER status may direct the regulation of endometrial injury repair [98]. Additional examples of hAEC treatment are presented in Table 1.

## Nanostructured scaffold

Tissue engineering has recently attracted considerable attention. Biomaterials science is now capable of directing cellular differentiation, as opposed to its emergence, when it was only a cell carrier tool [99]. In fact, through molding, biomaterials could be utilized for synthesizing three-dimensional scaffolds capable of promoting cell differentiation and/or proliferation for regeneration [100]. Stem cell activities are significantly affected by extracellular forces, micro-geometry, matrix nano-topography, and matrix stiffness as mechanical factors. Depending on their source of derivation, biomaterials could be classified into synthetic and natural polymers. Among natural scaffolds, we can refer to keratin, chitosan, alginate, silk fibroin, collagen, and de-cellularized tissues such as de-epithelialized human amniotic membrane [101]. Following the transplantation of BM-MSCs-loaded collagen, it was reported that MSCs are primarily located in the basal layer of regenerative endometrium although it should be mentioned that numerous cells migrate to the wound sites [102]. Interestingly, the injured endometrium prompted BM-MSCs arrangement such that they became capable of playing key roles in remodeling a new functional endometrium. Cells in the proximity of the implantation site of BM-MSCs/collagen constructs exhibited a greater amount of vascular endothelial growth factor VEGF, transforming growth factor (TGFb1), and insulin-like growth factor (IGF-1) compared with the spontaneous regeneration group and the collagen/PBS group. Such growth factors are necessary to the regeneration of the endometrial cycle [103]. Hyaluronic acid (HA) is reported to be utilized in regenerating the endometrium in several damaged models [104]. In fact, there exists a correlation between the endometrial receptivity and the HA level for pre-implanting embryos. This result is in line with the high HA concentration in the remodeling tissues [105].

Since HA can interact with the extracellular matrix and contributes to the matrix molecules, endometrial stromal cell-loaded HA hydrogels are presumably the most suitable candidates for endometrial regeneration. It has been suggested that HA receptors exist throughout each estrous cycle, even though their contents might differ from cycle to cycle [106]. HA/fibrin hydrogels have been synthesized utilizing concentrations of T optimized for accelerating cross-linking and facilitating the delivery of well-conditioned cells. Since such conditioning could exert effects on the efficiency of treatment, a study examined the effect of different T concentrations on stiffness for constructing the most suitable platform [107]. Further examples of scaffolds in this regard are provided in Table 1.

## Conclusion

As a global disease, infertility affects a great number of females and is both a social and medical issue. Given the vital role of endometrium in maternal health and reproduction, we believe that maintaining its physiological structure, eliminating its defects, and restoring it after injuries are of paramount importance. Likewise, stem cells have been the subject of many studies due to their effective functions. Therefore, we could deduce that stem cell therapy could be introduced for treating damaged tissues, cancer, and degenerative diseases as conditions with limited therapeutic options. Considering its recent advances, cell therapy has been proposed to be capable of treating numerous diseases including thin endometrium and AS. Various strategies have been introduced in stem cell therapy for boosting the survival of transplanted cells. In fact, there exist biomaterials such as nanostructure lipid carriers, hydrogels, and scaffolds, which are capable of promoting stem cell/drug delivery and thus improving the outcomes of stem cell therapy. Moreover, nano-engineered titanium implants could play vital parts in controlling stem cell/drug release. To regenerate the endometrium, we propose that the human umbilical cord mesenchymal seems to be the best clinical option as it is easily accessible, has rapid self-renewal features, is harvested abundantly through noninvasive procedures, and has low immunogenic effects. These characteristics are also common among other cell types such as PRP and hAEC. In our opinion, despite the advances in the field of cell therapy, major concerns still exist. For instance, it has been reported that endometrial stem cells are associated with the pathogenesis of many gynecological diseases (e.g., endometriosis, endometrial hyperplasia, and endometrial cancer). Other concerns as we believe include the fact that stem cells are capable of stimulating angiogenesis through the secretion of the growth factor. Overall, we could state that these novel therapies might actually enjoy numerous advantages over the traditional therapies in this regard. However, we understand that there still exists room for improvement, and much research is required to maximize the potentials of emerging novel therapies. Finally, with the advent of new cell therapies and the potential for infertility treatment, we are hopeful that many of the problems in this area will be resolved soon, especially in developing countries.

## Data Availability

Data sharing is not applicable to this article as no new data were created or analyzed in this study.

## Abbreviations

ART: Assisted reproductive technology
AS: Asherman syndrome
BMSCs: Bone marrow mesenchymal stem cells
EGF: Epidermal growth factor
EMT: Extra matrix transmission
EnMSCs: Endometrium mesenchymal stem cells
FET: Frozen-thawed embryo transfer
GCSF: Granulocyte colony-stimulating factor
GFS: Growth factors
GnRH: Gonadotropin-releasing hormone
GVHD: Graft-versus-host disease
hAECs: Human amniotic epithelial cells
HCG: Human chorionic gonadotropin
HRT: Hormone replacement therapy
IGF: Insulin-like growth factor
IUA: Intrauterine adhesion
MSCs: Mesenchymal derived stem cells
NK: Natural killer cell
PBMC: Peripheral blood mononuclear cell
PRP: Platelet-rich plasma
TGF: Transforming growth factor
Th: Tcell helper
TNF-α: Tumor necrosis factor-α
VEGF: Vascular endothelial growth factor
